# Circulating NOS3 Modulates Left Ventricular Remodeling following Reperfused Myocardial Infarction

**DOI:** 10.1371/journal.pone.0120961

**Published:** 2015-04-14

**Authors:** Simone Gorressen, Manuel Stern, Annette M. van de Sandt, Miriam M. Cortese-Krott, Jan Ohlig, Tienush Rassaf, Axel Gödecke, Jens W. Fischer, Gerd Heusch, Marc W. Merx, Malte Kelm

**Affiliations:** 1 Medical Faculty, Division of Cardiology, Pulmonology & Vascular Medicine, Heinrich-Heine-University, Düsseldorf, Germany; 2 Medical Faculty, Department of Cardiovascular Physiology, Heinrich-Heine-University, Düsseldorf, Germany; 3 CARID, Cardiovascular Research Institute Düsseldorf, Düsseldorf, Germany; 4 Medical Faculty, Institute of Pharmacology und Clinical Pharmacology, Heinrich Heine University, Cardiovascular Research Institute Düsseldorf (CARID), Düsseldorf, Germany; 5 Institute for Pathophysiology, West German Heart and Vascular Center Essen, University of Essen Medical School, Essen, Germany; 6 Department of Cardiology, Vascular Medicine and Intensive Care Medicine, Robert Koch Krankenhaus, Klinikum Region Hannover, Hannover, Germany; Virginia Commonwealth University Medical center, UNITED STATES

## Abstract

**Purpose:**

Nitric oxide (NO) is constitutively produced and released from the endothelium and several blood cell types by the isoform 3 of the NO synthase (NOS3). We have shown that NO protects against myocardial ischemia/reperfusion (I/R) injury and that depletion of circulating NOS3 increases within 24h of ischemia/reperfusion the size of myocardial infarction (MI) in chimeric mice devoid of circulating NOS3. In the current study we hypothesized that circulating NOS3 also affects remodeling of the left ventricle following reperfused MI.

**Methods:**

To analyze the role of circulating NOS3 we transplanted bone marrow of NOS3^−/−^ and wild type (WT) mice into WT mice, producing chimerae expressing NOS3 only in vascular endothelium (BC−/EC+) or in both, blood cells and vascular endothelium (BC+/EC+). Both groups underwent 60 min of coronary occlusion in a closed-chest model of reperfused MI. During the 3 weeks post MI, structural and functional LV remodeling was serially assessed (24h, 4d, 1w, 2w and 3w) by echocardiography. At 72 hours post MI, gene expression of several extracellular matrix (ECM) modifying molecules was determined by quantitative RT-PCR analysis. At 3 weeks post MI, hemodynamics were obtained by pressure catheter, scar size and collagen content were quantified post mortem by Gomori’s One-step trichrome staining.

**Results:**

Three weeks post MI, LV end-systolic (53.2±5.9μl;***p≤0.001;n = 5) and end-diastolic volumes (82.7±5.6μl;*p<0.05;n = 5) were significantly increased in BC−/EC+, along with decreased LV developed pressure (67.5±1.8mmHg;n = 18;***p≤0.001) and increased scar size/left ventricle (19.5±1.5%;n = 13;**p≤0.01) compared to BC+/EC+ (ESV:35.6±2.2μl; EDV:69.1±2.6μl n = 8; LVDP:83.2±3.2mmHg;n = 24;scar size/LV13.8±0.7%;n = 16). Myocardial scar of BC−/EC+ was characterized by increased total collagen content (20.2±0.8%;n = 13;***p≤0.001) compared to BC+/EC+ (15.9±0.5;n = 16), and increased collagen type I and III subtypes.

**Conclusion:**

Circulating NOS3 ameliorates maladaptive left ventricular remodeling following reperfused myocardial infarction.

## Introduction

Nitric oxide (NO) plays a central role in myocardial ischemia/reperfusion (I/R)-injury [1–3] and is constitutively produced within the endothelium and in several blood cell types by the isoform 3 of NO synthase (NOS3) e.g. in B- and T-lymphocytes [4], eosinophils [5], and in red blood cells (RBCs) [6, 7]. Recently we demonstrated that depletion of circulating NOS3 increases the size of reperfused myocardial infarction in a murine model [8].

Decreased NO during myocardial I/R results in increased formation of pro-inflammatory enzymes, cytokines and adhesion molecules [9], increased leukocyte rolling [10] and adhesion [11]. Impaired NO production may adversely affect left ventricular (LV) remodeling. LV remodeling is a chronic (mal-) adaptive process, characterized by excessive matrix restructuring, destruction of geometry, interstitial inflammation and fibrosis, progressive ventricular dilatation and deterioration in cardiac function leading to progressive heart failure and premature death [12–14].

It is the aim of the present study to evaluate the effects of depletion of circulating NOS3 on adverse LV functional and structural remodeling after reperfused myocardial infarction.

## Methods

### Animals

Male C57BL/6 wild type (WT) and NOS3−/−–mice (endothelial nitric oxide synthase) (C57BL/6.129/Ola-eNOStm) [15], were kept according to federal regulations. All procedures were performed in accordance with the national guidelines on animal care and were approved by the local Research Board for animal experimentation (LANUV = State Agency for Nature, Environment and Consumer Protection). Mice ranged in body weight from 20–25 g and in age from 8–10 weeks for bone marrow transplantation. Animals received a standard diet and water ad libitum.

### Chimerae (irradiation and bone marrow transplantation)

To analyze the effects of depletion of NOS3 in blood cells in a chronic model of myocardial I/R, we transplanted bone marrow from WT and NOS3−/− mice into WT mice, producing chimerae which either do (BC+/EC+) or do not carry NOS3 in blood cells (BC−/EC+) as described previously [8, 16]. (See **S1 File** for detailed information).

### Myocardial ischemia and reperfusion protocol

**Fig 1:**
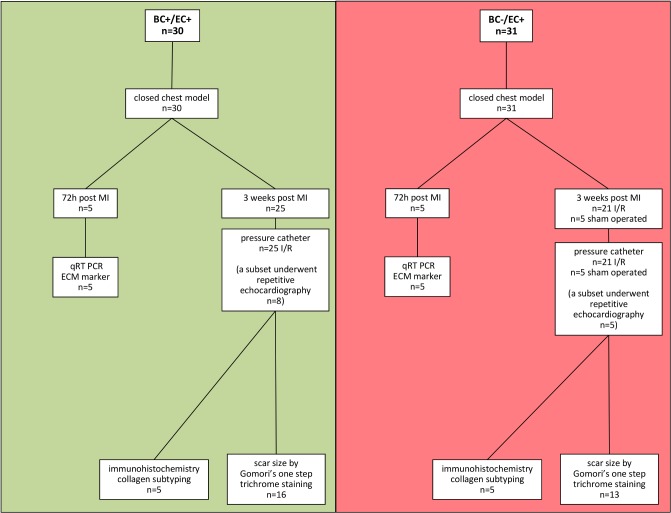
Flow chart of the presented study. In a closed chest model, animals were subjected to reperfused myocardial infarction. After 60 min of ischemia, animals were divided into two different groups: 1) 72 h post MI 2) 3 weeks post MI. Further analysis followed as depicted.

Six weeks after bone marrow transplantation a closed-chest model of reperfused myocardial infarction was utilized in order to reduce surgical trauma and consequent inflammatory reaction following I/R as compared to an open-chest model [8]. At 3 days post instrumentation myocardial infarction was induced by 60 min coronary occlusion. Coronary occlusion was achieved via gently pulling the applied suture tight until ST-elevation appeared on the ECG. After 60 minutes of ischemia, reperfusion was accomplished by cutting the suture close to the chest wall. Reperfusion was confirmed by resolution of ST-elevation. Reperfusion was performed for 3 weeks. We strictly adhered to ischemia induction between 8 am and 11 am to ensure equal I/R tolerance. (See **Fig. 1**and **S1 File** for detailed information)

### Echocardiography

Cardiac images were acquired applying a Vevo 2100 high-resolution ultrasound scanner with 18–38 MHz linear transducer (VisualSonics Inc.). Echocardiography was performed as previously described [17] pre MI, 24 hours, 4 days, 1 week, 2 weeks and 3 weeks post MI. Left ventricular (LV) end-systolic volumes (ESV), end-diastolic volumes (EDV) and LV ejection fraction (EF) were calculated, as previously described [8]. (See **S1**for detailed information)

### Invasive hemodynamic measurement of LV function

Invasive hemodynamics were assessed 3 weeks post myocardial infarction by a 1.4F Millar pressure catheter (SPR-839, Millar Instrument, Houston, TX, USA). Mice were anesthetized with ketamine (60mg/kg body weight) and xylazine (10mg/kg BW). For mean arterial pressure assessment the catheter was placed into the aorta ascendens. Thereafter, the catheter was placed into the left ventricle. The data were analyzed by IOX Software (EMKA) to obtain left-ventricular developed pressure (LVDP) and minimum and maximum rate of pressure change in the ventricle (dP/dtmin respectively dP/dtmax), as previously described [18].

### RNA extraction and quantitative RT-PCR analysis

Animals were sacrificed by cervical dislocation, and RNA was extracted from whole mouse hearts using mirVana miRNA Isolation Kit (Ambion) according to the manufacturer´s instructions. 1 μg RNA was transcribed to complementary DNA with the QuantiTect Reverse Transcription Kit (Qiagen) according to manufactures instructions.

Real-time PCR was performed in triplicate using the Applied Biosystems 7500 Fast Real-time PCR system (Applied Biosystems, Carlsbad, CA, USA) and TaqMan GenExpression Assays (Applied Biosystems) for *collagen type I* (Mm00801666_g1), *collagen type III* (Mm01254476_m1), *collagen type IV* (Mm01210125_m1), *matrix metalloproteinase-2* (Mm00439498_m1), *matrix metalloproteinase-9* (Mm00442991_m1), *tissue inhibitor of metalloproteinase-1* (Mm00441818_m1), *tissue inhibitor of metalloproteinase-2* (Mm00441825_m1), *tissue inhibitor of metalloproteinase-3* (Mm00441826_m1), *tissue inhibitor of metalloproteinase-4* (Mm01184417_m1) and *fibronectin-1* (Mm01256744_m1). *Gapdh* (glyceraldehyde-3-phosphate dehydrogenase; Mm99999915_g1) was chosen as endogenous loading control. The setup of reaction consisted of 10ng cDNA, Taqman primer set and Tagman Gene Expression Master Mix (Applied Biosystems; #4369016). PCR was performed according to the manufacturer’s instructions (standard run type), as previously described [18]. For *Biglykan* and *Decorin* quantitative real-time RT-PCR was performed with StepOnePlus Real-Time PCR System (Applied Biosystems) using Platinum SYBR Green qPCR SuperMix-UDG (InvitrogenTM Life Technologies Corporation). *Gapdh* was chosen as endogenous control, primer sequences are provided in **Table 1**.

**Table 1:** Primer sequences for mRNA expression assay.

Gene	forward	reverse
*Gapdh*	TGGCAAAGTGGAGATTGTTGCC	AAGATGGTGATGGGCTTCCCG
*Bgn*	CTGAGGGAACTTCACTTGGA	CAGATAGACAACCTGGAGGAG
*Dcn*	TAAAAGGTCGTGAAAATACAT	GAAGTCAAATAAGCCTCTCTG

### Assessment of scar size and wall thickness via Gomori’s one-step trichrome staining

Three weeks post myocardial infarction animals were sacrificed by cervical dislocation, and hearts were fixed in 4% formalin and embedded in paraffin. Cross sections of the heart (10 per mouse, 250 μm apart, up to the mitral valve) were obtained and stained with Gomori´s one-step trichrome staining. The fibrous area was determined in all sections using Diskus software (Hilgers) and expressed as percentage of total LV volume, as described previously [19]. (See **S1**for detailed information). Additionally, minimum wall thickness was measured in the infarcted and the remote myocardium 3 weeks post myocardial infarction.

### Immunohistochemistry—Content of collagen I, III and IV

Serial sections (3 per mouse, 250 μm apart) were stained to analyze the content of collagen I (rabbit polyclonal IgG collagen I, # ab292, abcam, UK), III (rabbit polyclonal IgG collagen III, # ab7778, abcam, UK), and IV (rabbit polyclonal IgG collagen IV, # ab19808, abcam, UK) in the infarcted area. As secondary antibody an anti-rabbit horseradish peroxidase conjugated IgG antibody (goat, polyclonal, Abbiotec, # 252237) was used. (See **S1**for detailed information)

### Statistical analysis

The results are given as mean ± standard error of the mean (SEM). For repeated measurements, data were analyzed by two-way ANOVA followed by Bonferroni’s post hoc test. Where indicated, an unpaired Student’s t test or one-way ANOVA followed by Bonferroni’s post hoc test was applied. p = 0.05 was set as the threshold of significance.

## Results

### LV function after reperfused myocardial infarction

**Fig 2:**
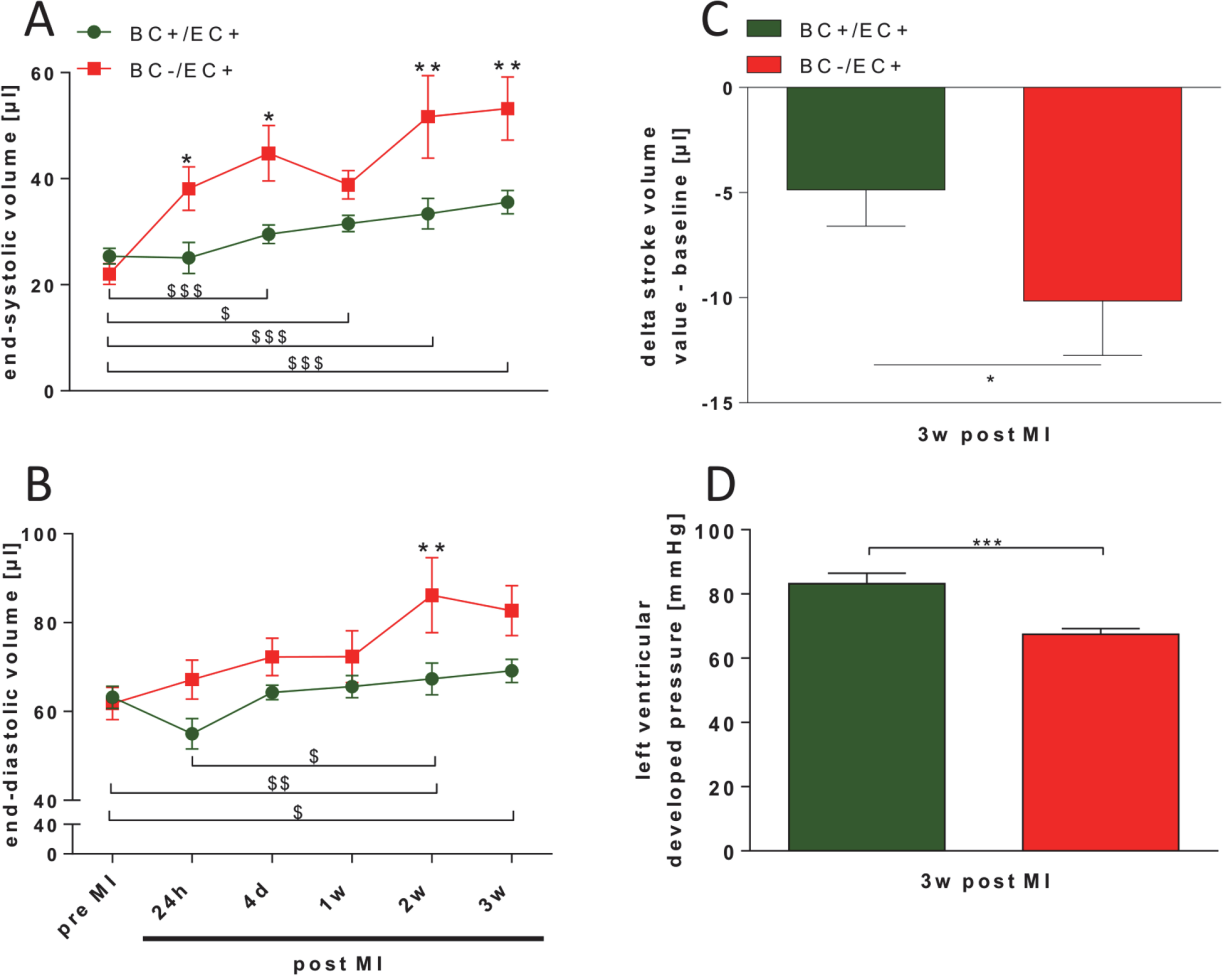
BC−/EC+ exhibited increased end-systolic and end-diastolic volume and decreased left ventricular function 3 weeks post MI. BC−/EC+ exhibited an increase in end-systolic (**A**) and end-diastolic volume (**B**), a significantly more pronounced decrease in stroke volume (**C**) (BC+/EC+ n = 8 and BC−/EC+ n = 5; two-way ANOVA and Bonferroni’s post hoc test or student’s t-test; * p<0.05, ** p≤ 0.01 BC+/EC+ vs. BC−/EC+; # p<0.05, ## p≤ 0.01, ### p≤ 0.001 BC+/EC+ at different time points; $ p<0.05, $$ p≤ 0.01, $ $ $ p≤ 0.001 BC−/EC+ at different time points), and decreased left ventricular developed pressure (**D**) 3 weeks post MI compared to BC+/EC+ (BC+/EC+ n = 24 and BC−/EC+ n = 18; student‘s t-test; ***p≤0.001).

While ejection fraction, end-systolic and end-diastolic volume did not differ between both groups before MI (see **Table A in S1 File**), BC−/EC+ exhibited decreased ejection fraction (***p≤ 0.001; n = 5), increased end-systolic volume (***p≤ 0.001; n = 5) and end-diastolic volume (*p<0.05; n = 5) compared to BC+/EC+ 3 weeks post MI (see **Table A in S1 File**). Accordingly, calculated stroke volume in BC−/EC+ (−10.16 ± 2.58μl; *p<0.05; n = 5) was less than in BC+/EC+ (−4.86 ± 1.72; n = 8) 3 weeks post MI (see **Fig. 2C**).

Over the time course of 3 weeks post MI, end-systolic volume was significantly increased in BC−/EC+ compared to BC+/EC+ (see **Fig. 2A**). Likewise, end-diastolic volume was significantly increased in BC−/EC+ at 2 and 3 weeks post MI compared to BC+/EC+ (See **Fig. 2B**). BC−/EC+ exhibited significantly decreased ejection fraction at 24h (43.4 ± 4.6%; *p<0.05; n = 5), 4 days (38.7 ± 4.6%; **p≤ 0.01; n = 5) and 3 weeks (36.4 ± 3.0%; *p<0.05; n = 5) post MI compared to BC+EC+ (24h: 55.2 ± 3.3%; 4 days: 54.1 ± 2.2%; 1 week: 51.8 ± 1.8%; 2 weeks: 50.8 ± 2.2; 3 weeks: 48.8 ± 1.7%; n = 8).

Three weeks post MI, LV function and mean arterial pressure were assessed via pressure catheter. LV developed pressure (***p≤0.001; n = 18), dp/dt_max_ (**p≤ 0.01; n = 18) and dP/dt_min_ (***p≤0.001; n = 18) were decreased in BC−/EC+ compared to BC+/EC+ (n = 24) 3 weeks post MI. (see **Fig. 2D** and **Table 2**). Heart rate did not differ between both groups (BC+/EC+: n = 25 vs. BC−/EC+: n = 21; n.s.) (see **Table 2**).

**Table 2:** BC−/EC+ exhibited decreased left ventricular function and decreased mean arterial pressure.

	3W post myocardial infarction		
	BC+/EC+	BC−/EC+	P-Value
Left ventricular developed pressure [mmHg]	83,2 ± 3,2	67,5 ± 1,8	p≤0,001
dp/dt_max_ [mmHg/s]	7485,6 ± 399,8	5933,1 ± 345,1	p≤ 0,01
dp/dt_min_ [mmHg/s]	−5634,1 ± 234,7	−4242,2 ± 185,8	p≤0,001
Heart rate [bpm]	570,0 ± 12,8	539,8 ± 18,0	ns
Mean arterial pressure [mmHg]	58,6 ± 1,7	43,3 ± 2,5	p≤0,001
Systemic vascular resistance [mmHg * min/ml]	3,241 ± 0,353	2,981 ± 0,5654	ns

LV developed pressure, dp/dt_max_ and dP/dt_min_ were decreased in BC−/EC+ compared to BC+/EC+ 3 weeks post MI. While systemic vascular resistance did not differ between both groups, BC−/EC+ exhibited decreased mean arterial pressure compared to BC+/EC+. (LV function: BC+/EC+ n = 24 and BC−/EC+ n = 18; mean arterial pressure: BC+/EC+ n = 25 and BC−/EC+ n = 21; student‘s t-test; ** p≤ 0.01; ***p≤0.001).

While systemic vascular resistance was not different between both groups 3 weeks post MI (BC+/EC+: n = 8 vs. BC−/EC+: n = 4; n.s.), BC−/EC+ exhibited decreased mean arterial pressure (***p≤0.001; n = 21;) compared to BC+/EC+ (n = 25) and sham operated BC−/EC+ (61.3 ± 2.9 mmHg) along with decreased stroke volume. (see **Table 2**).

### Expression pattern of fibrosis-related genes 72 h post reperfused myocardial infarction

**Fig 3:**
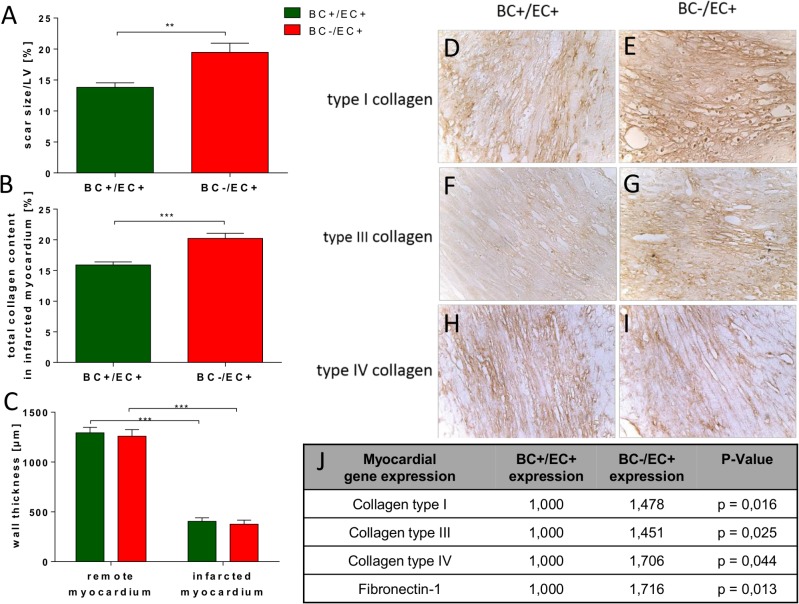
BC−/EC+ had increased scar size and total collagen content in the infarcted myocardium. BC−/EC+ exhibited increased scar size/left ventricle (**A**) and increased collagen content (**B**) in the infarcted myocardium. Minimum wall thickness (**C**) in infarcted myocardium was thinner in both groups than in remote myocardium. However, minimum wall thickness of infarcted myocardium did not differ between both groups. Subtyping of collagen type I (**D,E**), III (**F,G**) and IV (**H,I**) in the infarcted myocardium exhibited increased collagen type I (**E**) and III (**G**) in BC−/EC+ compared to BC+/EC+, while collagen type IV (**H,I**) was equal in both analyzed groups 3 weeks post MI (infarct size: BC+/EC+ n = 16 and BC−/EC+ n = 13; student‘s t-test; ** p≤ 0.01; total collagen: BC+/EC+ n = 16 and BC−/EC+ n = 13; student‘s t-test; *** p≤ 0.001; immunohistochemistry was performed with n = 5 per group). Myocardial gene expression 72 h post MI showed significantly up-regulated gene expression of collagen type I, III, IV and fibronectin-1 in BC−/EC+ compared to BC+/EC+. (n = 5 per group; *p< 0.05; analyzed by REST 2009 software (Qiagen)).

Seventy-two hours post myocardial MI, BC−/EC+ exhibited significantly up-regulated gene expression of *collagen type I* (*p = 0.016), *collagen type III* (*p = 0.025) and *collagen type IV* (*p = 0.044; BC+/EC+ n = 5, BC−/EC+ n = 4) throughout the heart compared to BC+/EC+. Likewise, *tissue inhibitor of metalloproteinase-1* (TIMP1; *p = 0.022), *fibronectin-1* (*p = 0.013) and *biglycan* (*p = 0,014) were significantly up-regulated in BC−/EC+, while *matrix metalloproteinase-9*, *tissue inhibitor of metalloproteinase-3* and *decorin* were only slightly but not significantly up-regulated throughout the heart compared to BC+/EC+. *Matrix metalloproteinase-2*, *tissue inhibitor of metalloproteinase-2* and *tissue inhibitor of metalloproteinase-4* did not differ between both groups. (see **Fig. 3J and Table B in S1 File**).

### Scar formation post reperfused myocardial infarction

BC−/EC+ hearts showed increased scar size/left ventricle (19.5 ± 1.5%, n = 13,** p≤ 0.01) compared to BC+/EC+ (13.8 ± 0.7%, n = 16) 3 weeks post MI (see **Fig. 3A**). BC−/EC+ had an increased total collagen content (20.2 ± 0.8%, n = 13, *** p≤ 0.001) in the infarcted myocardium 3 weeks post MI compared to BC+/EC+ (15.9 ± 0.5%, n = 16) (see **Fig. 3B**). Semi-quantitative immunohistochemistry indicated an increased content of collagen type I and III in BC−/EC+ (n = 5) compared to BC+/EC+ (n = 5) 3 weeks post MI, whereas the content of collagen IV did not differ in the infarcted myocardium between both groups 3 weeks post MI (see **Fig. 3 D-I**). Minimum wall thickness in remote (BC+/EC+ 1293.8 ± 53.9 μm vs. BC−/EC+ 1258.4 ± 67.6 μm; n = 12 per group; n.s.) and infarcted myocardium (BC+/EC+ 404.6 ± 35.4 μm vs. BC−/EC+ 376.3 ± 40.0 μm); n = 12 per group; n.s.) did not differ between both groups 3 weeks post MI. (see **Fig. 3C**).

## Discussion

The findings of the present study show that blood-borne NOS3 attenuates adverse left ventricular remodeling in reperfused myocardial infarction. These findings are associated with reduced infarct size and altered expansion and composition of the scar.

### Circulating NOS3 and left ventricular function post MI

In order to investigate the influence of circulating NOS3 on LV structure and function after reperfused MI, LV function and dimensions were analyzed in both groups by echocardiography and pressure catheter. LV developed pressure (LVDP), contractility (dP/dtmax), relaxation (dP/dtmin), as well as ejection fraction (EF) were reduced 3 weeks post MI. Furthermore, depletion of circulating NOS3 was associated with significantly increased end-systolic (ESV) and end-diastolic volume (EDV) 3 weeks post myocardial infarction. Ventricular dilatation is an important predictor of cardiac mortality [20]. The end-diastolic volume is a reliable indicator of left ventricular (LV) dilatation and frequently used to quantify the extent of adverse cardiac remodeling [21].

In mice the early phase of remodeling begins a few hours after MI and may persist for up to 2 weeks [22]. It is associated with different complex molecular, functional and structural changes. NO donors are able to diminish adverse remodeling as a result of infarction, and in consequence a reduction in NO bioavailability leads to adverse remodeling [23]. Thus, circulating NOS3 limits adverse remodeling following myocardial infarction likely through increased NO bioavailability during reperfusion after myocardial infarction.

### Circulating NOS3 and scar formation

Deletion of circulating NOS3 was associated with a significantly increased scar size 3 weeks post MI. The observed differences in scar formation underpin a potential role of blood-borne NOS3, likely facilitated by enhanced NO bioavailability. NO inhibits neutrophil adhesion [11], the release of ROS and leukocyte accumulation during early reperfusion [24]. As NO reduces leucocyte adhesion [25, 26], reduced inflammation is likely facilitated by increased NO bioavailability. Likewise, the benefits observed following hemoglobin substitution or enrichment of intact erythrocytes in the context of I/R might be—at least in part—mediated by increased NO bioavailability [27, 28]. We cannot rule out that, apart from the deletion of circulating NOS3, other major ROS dependent signaling cascades might have been affected by bone marrow transplantation experiments and thus additionally might have contributed to the observed healing processes and formation of scar post reperfused MI.

To further elucidate the structural remodeling and thereby the qualitative characteristics of the healing wound we assessed the collagen content of the myocardial scar 3 weeks post MI. The larger infarcted myocardial tissue area was characterized by increased collagen content in the infarcted myocardium of animals with depleted circulating NOS3. The time point “3 weeks post MI” is frequently regarded as the “stable” phase in the remodeling process termed “scar maturation phase” [29]. It is critical that a strong, mature scar is formed as early as possible since the structurally weakened infarct area is subject to expansion during the proliferative phase and vulnerable to rupture [30]. Preservation of the extracellular matrix and collagen deposition at the site of myocyte necrosis are essential for structural stability of the infarcted heart. Excessive collagen degradation and impaired fibrous tissue formation may reduce the tensile strength of the necrotic zone and lead to enhanced infarct expansion [31, 32]. On the other hand, increased fibrosis stiffens the left ventricle and impairs its diastolic filling [33].

The major fibrillar collagens are type I and III in the cardiac extracellular collagen matrix (ECM) [34], while collagen type IV is located in the basal membrane of the cardiomyocytes [35, 36]. An increased proportion of the collagens I, II and IV is associated with adverse remodeling and LV dysfunction [33], and collagen types I and III were also up-regulated in a rat model of I/R [37]. In our study, the depletion of circulating NOS3 was associated with an increased deposition of collagen I and III in the infarcted myocardium. Therefore, circulating NOS3 appears to modulate the expansion and quality of the myocardial scar and thus to affect LV function and structure.

In our setup, following myocardial ischemia biglycan, decorin and Timp1 mRNA levels are increased in BC−/EC+. It is tempting to speculate, that the levels are increased as a response to the augmented myocardial injury and, as a consequence, stabilize the collagen rich scar in this group. Biglycan and decorin are involved in the organisation of collagen fibril networks especially collagen 1 and 3 [38, 39] and biglycan mRNA levels have been demonstrated to be increased in the infarcted zone after MI [40]. Biglycan deficiency leads to disturbed collagen deposition, disturbed remodeling and hemodynamic insufficiency after myocardial infarction with subsequent ventricular rupture [41, 42]. Furthermore, biglycan is known to protect cardiomyocytes against hypoxia/ reoxygenation injury by activation of NOS3 [43]. In this context, the impact of biglycan on NOS3 in BC−/EC+ would be expected to be decreased due to missing circulating NOS3.

### Study limitation

By applying our chimera model, we cannot determine to what extent the changes in left ventricular remodeling are due to the modulation of infarct size in the first 24 h after ischemia, and to what extent circulating NOS3 has an additional impact on the functional and structural LV remodeling and scar composition process. However, no model is presently available that allow an inhibition of circulating NOS3 beginning no sooner than e. g. 24h post induction of ischemia to exclusively analyze the impact of circulating NOS3 in the late remodeling process. This hold all the more true, as neither an inhibitor nor a model is known which would allow answering this question without affecting the running experiment and especially left ventricular remodeling itself. Accordingly, our results provide a clear gain in knowledge, because they show for the first time, that a lack of circulating NOS3 leads initially not only to an increased infarct and decreased left ventricular function, but results also in modulation of the quantity and quality of myocardial scar and consequently adverse functionally remodeling.

In conclusion, circulating NOS3 attenuates maladaption in the healing process of reperfused myocardial infarction through preserved LV dimension and function accompanied by altered scar formation. Our findings suggest that modulating circulating NOS3 might be a promising therapeutic approach in attenuating LV adverse remodeling following myocardial infarction.

## Supporting Information

S1 File Supplement information is provided in a separate file named “S1 File”. **Table A—BC−/EC+ exhibited decreased ejection fraction and increased end-systolic and end-diastolic volumes (Table A is included in the “S1 File”).** Three weeks post MI BC−/EC+ had decreased ejection fraction, increased end-systolic and end-diastolic volumes compared to BC+/EC+ (data are shown as mean ± SEM; BC+/EC+ n = 8 and BC−/EC+ n = 5; presented data were tested with two-way ANOVA and Bonferroni’s post hoc test; * p<0.05; ** p≤ 0.01, *** p≤0.001). **Table B—BC−/EC+ had modulated myocardial gene expression 72 h post MI (Table B is includes in the “S1 File”).** Myocardial gene expression 72 h post MI exhibited significantly up-regulated gene expression of TIMP1 and Biglycan in BC−/EC+ animals, while MMP9, TIMP3 and Decorin were only slightly but not significantly up-regulated compared to BC+/EC+. MMP2, TIMP2 and TIMP4 did not differ between both groups. (BC+/EC+ n = 5, BC−/EC+ n = 4; *p<0.05; myocardial gene expression was analyzed by REST 2009 software (Qiagen)).
(DOCX)
