# Supplementary material for: Circulating NOS3 Modulates Left Ventricular Remodeling following Reperfused Myocardial Infarction
Source: PLoS One. 2015 Apr 14;10(4):e0120961. doi: 10.1371/journal.pone.0120961 (PMC4397096; doi:10.1371/journal.pone.0120961)
Supplement: S1 File — Table A—BC−/EC+ exhibited decreased ejection fraction and increased end-systolic and end-diastolic volumes (Table A is included in the “S1 File”). Three weeks post MI BC−/EC+ had decreased ejection fraction, increased end-systolic and end-diastolic volumes compared to BC+/EC+ (data are shown as mean ± SEM; BC+/EC+ n = 8 and BC−/EC+ n = 5; presented data were tested with two-way ANOVA and Bonferroni’s post hoc test; * p<0.05; ** p≤ 0.01, *** p≤0.001). Table B—BC−/EC+ had modulated myocardial gene expression 72 h post MI (Table B is includes in the “S1 File”). Myocardial gene expression 72 h post MI exhibited significantly up-regulated gene expression of TIMP1 and Biglycan in BC−/EC+ animals, while MMP9, TIMP3 and Decorin were only slightly but not significantly up-regulated compared to BC+/EC+. MMP2, TIMP2 and TIMP4 did not differ between both groups. (BC+/EC+ n = 5, BC−/EC+ n = 4; *p<0.05; myocardial gene expression was analyzed by REST 2009 software (Qiagen)). (DOCX) [file pone.0120961.s001.docx]

**Circulating NOS3 modulates left ventricular remodeling following reperfused myocardial infarction**

Simone Gorressen*^1^, Manuel Stern*^1^, Annette M. van de Sandt*^1^_,_ Miriam M. Cortese-Krott^1^, Jan Ohlig^1^, Tienush Rassaf^1^, Axel Gödecke^2,3^, Jens W. Fischer^3,4^, Gerd Heusch^5^_,_ Marc W. Merx^1,6^ & Malte Kelm^1,3^

^1^ Medical Faculty, Division of Cardiology, Pulmonology & Vascular Medicine, Heinrich-Heine-University, Düsseldorf, Germany;

^2^ Medical Faculty, Department of Cardiovascular Physiology, Heinrich-Heine-University, Düsseldorf, Germany;

^3^ CARID, Cardiovascular Research Institute Düsseldorf

^4^ Medical Faculty, Institute of Pharmacology und Clinical Pharmacology, Heinrich Heine University, Cardiovascular Research Institute Düsseldorf (CARID), Düsseldorf, Germany;

^5^ Institute for Pathophysiology, West German Heart and Vascular Center Essen, University of Essen Medical School, Essen, Germany

^6^ Department of Cardiology, Vascular Medicine and Intensive Care Medicine, Robert Koch Krankenhaus, Klinikum Region Hannover, Hannover, Germany

***S.G., M.S. and AM.S. contributed equally**

**Address for correspondence:**

Malte Kelm M.D.

Department of Medicine

Division of Cardiology, Pneumology and Angiology

Moorenstrasse 5, 40225 Düsseldorf, Germany

Phone: +49 (0) 211- 8118801, Fax: +49 (0) 211- 8118812

Email: malte.kelm@med.uni-duesseldorf.de

**Supporting information (SI)**

**Methods**

Chimerae (irradiation and bone marrow transplantation)

We transplanted WT and NOS3^-/-^ bone marrow in WT mice, producing chimerae expressing NOS3 only in vascular endothelium (BC-/EC+) or in both (BC+/EC+). Twenty-four hours prior to the respective bone marrow transplant a hyper-fractionated irradiation was performed. The mice were treated in an interval of 4 hours, each with 6.5 Gy. Per irradiation the dose of 6.5 Gy was divided into two single doses of 3.25 Gy. We started with an irradiation from anterior to posterior with 3.25 Gy followed by irradiation of posterior to anterior, also with 3.25 Gy. Animals received water supplemented with Borgal (24%, 1 ml/l; sulfadoxinum 200 mg, trimethoprimum 40 mg) ad libitum directly after the first irradiation. Three weeks after successful bone marrow transplantation Borgal treatment was discontinued. To avoid any influence of antibiotics on the following experiments, the animals received an additional recovery time of further 3 weeks (total recovery time 6 weeks). Furthermore, there was no necessity to treat animal with buprenorphine to minimize animal suffering. Twenty-four hours after hyper-fractionated irradiation of the recipient mice, bone marrow transplantation was performed. The bone marrow was isolated from tibia and femur. The donor animal was anesthetized with ketamine (60mg/kg body weight) and xylazine (10mg/kg BW). After isolation of the bones they were rinsed with physiological buffer solution (PBS). The syringes were provided with a 0.22 micron sterile filter (Millipore) and a 27G needle (BD). The PBS cell mixture was collected. The cells were re-suspended thoroughly and then placed on a filter 40 μM (BD). The cell suspension was subsequently centrifuged for 10 min at 300 g at room temperature. The supernatant was discarded, and the cells were suspended in 10 ml of PBS. An aliquot (20 μl) of the cell suspension was taken for cell counting, and the remaining cell suspension again centrifuged at 300 g for 10 min at room temperature. After centrifugation cells were re-suspended to 2-5*10^6^/100μl. Bone marrow cells were given to each recipient mouse by intra-cardiac injection. Therefore, the recipient mouse was anesthetized using isoflurane anesthesia. Subsequently, the application of the cell suspension was performed using a 30 G needle. Successful intra-cardiac puncture was checked by aspirating a small amount of blood before and after the application.

Myocardial ischemia and reperfusion protocol

A closed-chest model of reperfused MI was utilized in order to exclude that any inflammatory reaction following reperfused MI was due to the surgical trauma itself [[38](#_ENREF_38)]. Mice were anesthetized by intra-peritoneal injection of ketamine (60mg/kg BW) and xylazine (10mg/kg body weight). After anesthesia was sufficient (confirmed by squeezing the paws), animals were intubated and connected to a rodent ventilator (Uno Microventilator). The mice were ventilated with a tidal volume of 200 μl at a rate of 140 strokes/min, with a mixture of two thirds air, one third oxygen and isoflurane 2.0-2.5 Vol.% (Forene ®, Abbott GmbH, Germany). Mice were placed in a supine position on a warmed plate. Body temperature was maintained at 37°C, and electrocardiography (ECG) (Hugo Sachs Apparatus) was monitored. After a left lateral thoracotomy between the third and fourth rib, the pericardium was dissected and a 7-0 surgical suture was cautiously passed underneath the left anterior descending coronary artery (LAD) at a position 1 mm from the tip of the left auricle. The 7-0 prolene suture was cut at the needle site, and both ends were threaded through a 1 mm section of PE-10 tubing, forming a loose snare around the LAD. Both ends of the suture were tightened to confirm the correct position of the suture (blanching of the apex and change in ECG). Each end of the suture was then threaded through the end of a size 3 Kalt suture needle (Fine Science Tools), exteriorized to the left side of the thorax and formed into a loop by knotting each end to the other. The loop was left in the subcutaneous tissue. The chest was closed with four interrupted stitches utilizing 6-0 suture. Anesthesia was turned off while closing the skin. After mice regained spontaneous breathing they were extubated and allowed to breathe 100% O_2_. Animal received buprenorphine (0.05 – 0.1mg/kg, s.c.) every 8 hours for first 3 days. At 3 days post instrumentation, the animals were re-anesthetized by mask inhalation of isoflurane 2.0 Vol.% and a mixture of one third oxygen and two thirds room air. Mice were placed in a supine position on a warmed plate. ECG was monitored to document ST-segment elevation. The skin was reopened and after dissecting the loop, both ends of the applied suture were gently pull tight until ST-elevation appeared on the ECG. After 60 minutes of ischemia, reperfusion was accomplished by cutting the suture close to the chest wall. Reperfusion was confirmed by resolution of ST-elevation. The skin was closed again. Reperfusion was performed for 3 weeks. Animal received buprenorphine (0.05 – 0.1mg/kg, s.c.) every 8 hours for one day. We strictly adhered to ischemia induction between 8 am and 11 am to ensure equal I/R tolerance.

Echocardiography

Cardiac images were acquired using a Vevo 2100 high-resolution ultrasound scanner with 18-38 MHz linear transducer (VisualSonics Inc.). Echocardiography was performed under slight mask anesthesia by an inhaled mixture of 1.5% (v/v) isoflurane and 100% oxygen. ECGs were obtained with built-in ECG electrode-contact pads. Body temperature was maintained at 37 °C by a heating pad. All hair was removed from the chest using a chemical hair remover (Veet). Aquasonic 100 gel (Parker Laboratories, Hellendoorn, Netherlands) was applied to the thorax surface to optimize the visibility of the cardiac chambers. Parasternal long-axis and short-axis views were acquired. Left ventricular (LV) end-systolic and end-diastolic volumes (ESV and EDV) were calculated by identification of frames with maximal and minimal cross-sectional area and width. The system software employs a formula based on a cylindrical-hemiellipsoid model [[39](#_ENREF_39)]. LV ejection fraction (LVEF) was calculated from volume data. A single ultrasound session ranged from 15 to 30 min per mouse**.**

Assessment of scar size via Gomori’s one-step trichrome staining (IS)

Three weeks post MI the animals were sacrificed by cervical dislocation, and hearts were excised and rinsed in 0.9% physiological saline. The extracted hearts were fixed overnight in 4% formalin, dehydrated by an ascending alcohol series (70%, 80%, 90%, 96% and 100%) and embedded in paraffin. Every 250 µm ten 5 µm thick sections were made (up to the mitral valve). The sections were air-dried for 24 h. Assessment of scar size was performed via Gomori 's one-step trichrome staining. The sections were deparaffinized twice in xylene for 15 min, and finally a descending alcohol series (100 %, 96%, 70%) was performed. Sections were treated in Bouin's solution (Sigma) at 58° C for 15 min. After 5 min rinsing under running water sections were treated with Weigert's iron hematoxylin A and B for 5 min (1:1, Sigma) for nuclear staining. The sections were rinsed again for 5 min with running water and stained 25 min with Gomori 's staining solution ( 0.1281 mmol/l chromotrope 2R; 0.00566 mmol/l aniline blue; 166.5 mmol/l of glacial acetic acid , 0.00277 mmol/l phosphotungstic acid).

After brief rinse with water, the sections were rinsed 2 x 2 min in 0.5 % acetic acid, followed by an ascending alcohol series and treatment in xylene (2 x 5 min). The sections were covered with mounting medium Vitro Clud (R. Langenbrinck) and documented using a Leica microscope and the imaging software Diskus [[40](#_ENREF_40)]. The fibrous area was determined in all sections and expressed as percentage of total LV volume, as previously described [[19](#_ENREF_19)]. Collagen content was calculated in serial sections (3 per mouse, 250 μm apart) as the positively stained area percentage of the fibrous area using the software Image J.

Immunohistochemistry - Content of collagen I, III and IV

Serial sections (3 per mouse, 250 μm apart) were stained to analyze the content of collagen I (rabbit polyclonal IgG collagen I, # ab292, abcam, UK), III (rabbit polyclonal IgG collagen III, # ab7778, abcam, UK) and IV (rabbit polyclonal IgG collagen IV, # ab19808, abcam, UK) in the infarcted area. As secondary antibody an anti-rabbit horseradish peroxidase conjugated IgG antibody (goat, polyclonal, Abbiotec, # 252237) was used. The sections were deparaffinized with xylene and rehydrated by a descending alcohol series (100 %, 96 %, 70 %). Subsequently, the sections were boiled for 20 min in citrate buffer for unmasking and cooled slowly down for 20 min. The sections were washed in PBS for 5 min., rinsed and incubated for 5 min with 3% H_2_O_2_ in a humid chamber at room temperature to inactivate the endogenous peroxidase, then again rinsed for 5 min in PBS and treated with blocking solution (1% BSA) for 1h at room temperature. The incubation with the relevant primary antibody took place overnight at 4° C. After rinsing with PBS for 5 min, the incubation with the secondary antibody was performed for 1 h at room temperature. As substrate 3,3 diaminobenzidine (Vector Laboratoris) was used. After ascending alcohol series (70 %, 96 %, 100 %, xylene) the sections were covered with mounting medium Vitro Clud (R. Langenbrinck ), documented using a Leica microscope, and the intensity and the distribution of the color were visually semi-quantitatively estimated.

Table A – BC-/EC+ exhibited decreased ejection fraction and increased end-systolic and end-diastolic volumes

Three weeks post MI BC-/EC+ had decreased ejection fraction, increased end-systolic and end-diastolic volumes compared to BC+/EC+ (data are shown as mean ± SEM; BC+/EC+ n=8 and BC-/EC+ n=5; presented data were tested with two-way ANOVA and Bonferroni’s post hoc test; * p<0.05; ** p≤ 0.01, *** p≤0.001).

|  | **Pre myocardial infarction** | | | **3W post myocardial infarction** | | | | |
| --- | --- | --- | --- | --- | --- | --- | --- | --- |
|  | **BC+/EC+** | **BC-/EC+** |  | **BC+/EC+** |  | **BC-/EC+** |  |  |
| n | 8 | 5 | BC+/EC+ vs  BC-/EC+  pre MI | 8 | Pre MI  vs  3w post  MI | 5 | Pre MI  vs  3w post MI | BC+/EC+ vs  BC-/EC+  3w post MI |
| B-mode variables |  |  | P-Value |  | P- Value |  | P-Value | P-Value |
| Ejection fraction (%) | 59,9±1,2 | 64,6±1,6 | ns | 48,8±1,7 | p≤0,001 | 36,4±3,0 | p≤0,001 | p≤0,001 |
| End-systolic volume (µl) | 25,38±1,5 | 22,0±1,9 | ns | 35,6±2,2 | P<0,05 | 53,2±5,9 | p≤0,001 | p≤0,001 |
| End-diastolic volume (µl) | 63,2±2,5 | 61,8±3,6 | ns | 69,1±2,6 | ns | 82,7±5,6 | p≤0,01 | P<0,05 |

Table B – BC-/EC+ had modulated myocardial gene expression 72 h post MI

Myocardial gene expression 72 h post MI exhibited significantly up-regulated gene expression of TIMP1 and Biglycan in BC-/EC+ animals, while MMP9, TIMP3 and Decorin were only slightly but not significantly up-regulated compared to BC+/EC+. MMP2, TIMP2 and TIMP4 did not differ between both groups. (BC+/EC+ n=5, BC-/EC+ n=4; *p<0.05; myocardial gene expression was analyzed by REST 2009 software (Qiagen)).

| **Myocardial**  **gene expression** | **BC+/EC+**  **expression** | **BC-/EC+**  **expression** | **P-Value** |
| --- | --- | --- | --- |
| *Biglycan* | 1,000 | 1,471 | p =0,014 |
| *Decorin* | 1,000 | 1,285 | p = 0,095 |
| *Matrix metalloproteinase-2 (Mmp2)* | 1,000 | 1,241 | p = 0,259 |
| *Matrix metalloproteinase-9 (Mmp9)* | 1,000 | 1,524 | p = 0,058 |
| *Tissue inhibitor of metalloproteinase-1 (Timp1)* | 1,000 | 1,478 | p = 0,022 |
| *Tissue inhibitor of metalloproteinase-2 (Timp2)* | 1,000 | 1,132 | p = 0,389 |
| *Tissue inhibitor of metalloproteinase-3 (Timp3)* | 1,000 | 1,434 | p = 0,260 |
| *Tissue inhibitor of metalloproteinase-4 (Timp4)* | 1,000 | 0,918 | p = 0,766 |
